# Both *cis* and *trans* Activities of Foot-and-Mouth Disease Virus 3D Polymerase Are Essential for Viral RNA Replication

**DOI:** 10.1128/JVI.00469-16

**Published:** 2016-07-11

**Authors:** Morgan R. Herod, Cristina Ferrer-Orta, Eleni-Anna Loundras, Joseph C. Ward, Nuria Verdaguer, David J. Rowlands, Nicola J. Stonehouse

**Affiliations:** a School of Molecular and Cellular Biology, Faculty of Biological Sciences and Astbury Centre for Structural Molecular Biology, University of Leeds, Leeds, United Kingdom; b Molecular Biology Institute of Barcelona (CSIC), Barcelona Science Park (PCB), Barcelona, Spain; University of Pittsburgh School of Medicine

## Abstract

The Picornaviridae is a large family of positive-sense RNA viruses that contains numerous human and animal pathogens, including foot-and-mouth disease virus (FMDV). The picornavirus replication complex comprises a coordinated network of protein-protein and protein-RNA interactions involving multiple viral and host-cellular factors. Many of the proteins within the complex possess multiple roles in viral RNA replication, some of which can be provided in *trans* (i.e., via expression from a separate RNA molecule), while others are required in *cis* (i.e., expressed from the template RNA molecule). *In vitro* studies have suggested that multiple copies of the RNA-dependent RNA polymerase (RdRp) 3D are involved in the viral replication complex. However, it is not clear whether all these molecules are catalytically active or what other function(s) they provide. In this study, we aimed to distinguish between catalytically active 3D molecules and those that build a replication complex. We report a novel nonenzymatic *cis*-acting function of 3D that is essential for viral-genome replication. Using an FMDV replicon in complementation experiments, our data demonstrate that this *cis*-acting role of 3D is distinct from the catalytic activity, which is predominantly *trans* acting. Immunofluorescence studies suggest that both *cis*- and *trans*-acting 3D molecules localize to the same cellular compartment. However, our genetic and structural data suggest that 3D interacts in *cis* with RNA stem-loops that are essential for viral RNA replication. This study identifies a previously undescribed aspect of picornavirus replication complex structure-function and an important methodology for probing such interactions further.

**IMPORTANCE** Foot-and-mouth disease virus (FMDV) is an important animal pathogen responsible for foot-and-mouth disease. The disease is endemic in many parts of the world with outbreaks within livestock resulting in major economic losses. Propagation of the viral genome occurs within replication complexes, and understanding this process can facilitate the development of novel therapeutic strategies. Many of the nonstructural proteins involved in replication possess multiple functions in the viral life cycle, some of which can be supplied to the replication complex from a separate genome (i.e., in *trans*) while others must originate from the template (i.e., in *cis*). Here, we present an analysis of *cis* and *trans* activities of the RNA-dependent RNA polymerase 3D. We demonstrate a novel *cis*-acting role of 3D in replication. Our data suggest that this role is distinct from its enzymatic functions and requires interaction with the viral genome. Our data further the understanding of genome replication of this important pathogen.

## INTRODUCTION

The Picornaviridae family of small, single-stranded positive-sense RNA viruses contains approximately 30 genera and includes many important human and animal pathogens. Poliovirus (PV) is the prototype of the enterovirus genus, which also includes the coxsackieviruses and the rhinoviruses. Other well-studied genera are the cardioviruses and the aphthoviruses, such as foot-and-mouth disease virus (FMDV). FMDV is the causative agent of foot-and-mouth disease, a highly infectious disease of cloven-hooved animals. Infection presents as an acute systemic disease with lesions on the feet and mouth; however, under certain circumstances the virus can adopt an asymptomatic carrier state. The virus is endemic in many regions of the world and can cause major outbreaks in domestic livestock with significant economic losses. Although vaccines are available, disease control is often complicated by high antigenic variability, transmissibility, and infectivity combined with the problem of asymptomatic carrier animals.

The FMDV RNA genome contains a single open reading frame flanked by both 5′ and 3′ untranslated regions (UTRs) (1). The FMDV 5′ UTR contains at least five discrete elements, including a type II internal ribosome entry site (IRES) (2–4) and the “*cis*-acting replicative element” or CRE (5). Additional elements of unknown function include a large 5′ stem-loop termed the S fragment, a polypyrimidine tract of variable length, and various numbers of RNA pseudoknots (1, 5–8). The smaller 3′ UTR contains two RNA stem-loops upstream of a 3′ poly(A) tail, both of which have been shown to have roles in viral RNA replication (9, 10).

Translation of the genome yields a single polyprotein which is co- and posttranslationally proteolytically processed to produce the structural and nonstructural (NS) proteins. Initial processing of the polyprotein generates four primary products: L^pro^ and the P1-2A, 2BC, and P3 precursors. L^pro^ is self-proteolytically processed at its C-terminal boundary with P1 (11). Release of P1-2A from the remaining polyprotein occurs cotranslationally through a 2A-mediated ribosome-skipping mechanism (12–16). Subsequent 3C/3CD-mediated proteolysis of P1-2A generates three structural proteins, 1AB, 1C, and 1D. 1AB is cleaved via an unknown mechanism into fully mature 1A and 1B during virion maturation (17–19). Intermediate polyprotein precursors (2BC and P3) undergo 3C/3CD-mediated proteolysis to produce the mature viral RNA replication proteins (20). Final processing of P3 is thought to occur through both major and minor pathways via a differential set of intermediary precursors to ultimately produce the 3A transmembrane protein (21–23), three nonidentical tandem copies of the primer polypeptide 3B (3B_1–3_) (24–28), the major viral protease 3C (29, 30), and the RNA-dependent RNA polymerase (RdRp) 3D (also known as 3D^pol^) (31). Due to the limited coding capacity of small RNA viruses, several key NS polyprotein precursors perform distinct functions in the viral life cycle. These include the relatively stable precursor proteins 3AB_1–3_ (3AB in PV) and 3CD (32–34). In addition, many viral proteins have multifunctional roles in viral RNA replication, some of which can be supplied in *trans* (i.e., from a separate RNA molecule), while others are required in *cis* (i.e., from within the same RNA molecule) (35–40).

Viral RNA replication is thought to occur within membrane-associated replication factories known as replication complexes, where the mature NS proteins together with some precursor proteins are thought to localize. Several RNA elements are also known to be essential for viral RNA replication, such as the CRE. In FMDV, this is located within the 5′ UTR, whereas in other picornaviruses this element can be found within the polyprotein-coding sequence (5, 41, 42). Both intracellular and *in vitro* studies using FMDV or PV have elucidated some of the interactions within the replication complex. The crystal structures of 3D alone and in complex, e.g., with RNA and the primer peptide 3B (also known as VPg) have shed light on the mechanism of RNA polymerization (31, 43–48). VPg undergoes 3D-mediated uridylylation on the third tyrosine residue to form VPg-pUpU, which subsequently acts as the primer for both positive- and negative-strand RNA synthesis (49, 50). The template for VPg uridylylation is the CRE RNA stem-loop (5, 51). However, there is conflicting evidence with PV as to whether the substrate for 3D-mediated VPg uridylylation is the precursor protein 3AB, 3BC, or 3BCD (52, 53). Under certain circumstances, the CRE and 3D can be supplied in *trans*, suggesting that the uridylylation mechanism is not entirely *cis* dependent (36, 39, 40, 54–57). In contrast to 3D, the essential 3CD precursor of PV has no polymerase activity but performs an independent function in viral RNA replication, most likely involving interaction with a structured RNA element in the 5′ UTR known as the cloverleaf (58–65). In aphthoviruses, such as FMDV, the cloverleaf RNA element is replaced by an alternative RNA stem-loop structure, the S fragment, located at the same genomic position and hypothesized to provide the same function (8, 66, 67).

Assembly of the replication complex is a dynamic process that is not fully characterized. The current models are thought to require genomic circularization mediated by a ribonucleoprotein complex formed between the 5′ and 3′ UTRs and requiring both viral and host proteins (37, 58, 59, 65, 68–73). In PV, this likely involves at least 3CD and poly(rC)-binding proteins 1 and 2 (PCBP1 and -2) at the 5′ UTR, binding to the cloverleaf and IRES, together with poly(A)-binding protein (PABP) at the 3′ UTR. In FMDV, there is evidence that PCBP2 binds the IRES, forming a complex with proteins at the 3′ UTR, e.g., PABP. A recent biochemical study with PV has provided *in vitro* evidence to suggest that 3D cannot assemble into the replication complex alone but is initially assembled as part of a larger precursor, which is subsequently processed to the active 3D component (74). Furthermore, the data from this study also suggest subsequent recruitment of additional P3 molecules (or a smaller precursor thereof) to supply active 3D and/or VPg to the replication complex. However, it is not entirely clear whether the same molecule of P3 provides functional molecules of both 3D and VPg. These observations support previous studies implicating the involvement of multiple molecules of 3D/3CD in viral RNA replication (75–79) and the observation that P3 was required to recover replication-defective mutations in 3A and a VPg linkage mutant (57, 80). However, it is essential to determine whether interactions deduced from *in vitro* cell-free studies truly reflect viral RNA replication within the cell.

In this study, we have investigated differential functions of 3D in the context of FMDV subgenomic replicons. We have exploited random mutagenesis to identify replication-defective 3D point mutations in specific functional regions of the polymerase and introduced these into replicons encoding different reporters for use in complementation experiments. Although all of the mutations allowed complete P3 processing, only those blocking 3D catalytic activity were recovered in *trans* using replication-competent “helper” transcripts. In contrast, all 3D mutations to catalytic and noncatalytic residues were recovered by transcripts that were replication deficient due to mutations in essential RNA elements. This implies a separate RNA binding *cis*-acting role of 3D in viral RNA replication. X-ray crystallographic studies showed that the *cis*-acting mutations did not disrupt the structure of 3D but suggested that they reduced RNA binding affinity. Finally, we suggest a model for how the different *cis* and *trans* actions of 3D contribute toward replication complex assembly.

## MATERIALS AND METHODS

### Cell lines.

BHK-21 cells obtained from the ATCC (LGC Standard) were maintained in Dulbecco's modified Eagle's medium with glutamine (Sigma-Aldrich) supplemented with 10% fetal calf serum (FCS), 50 U/ml penicillin, and 50 μg/ml streptomycin as previously described (56). BSR-T7 cells (a kind gift from John Barr, University of Leeds) were maintained in Dulbecco's modified Eagle medium with glutamine (Sigma-Aldrich) supplemented with 10% FCS, 50 U/ml penicillin, 50 μg/ml streptomycin, and 500 μg/ml G418 (Life Technologies).

### Plasmid construction.

The FMDV replicon plasmids pRep-ptGFP (where GFP is green fluorescent protein) and pRep-mCherry along with the equivalent polymerase knockout controls 3D-GNN have already been described (56). To introduce individual replication-defective 3D insertions, the corresponding EcoRI-BamHI fragments were removed from previously reported pGFP-PAC replicons harboring the corresponding insertions and introduced into EcoRI-BamHI-digested pRep-mCherry. Individual 3D coding changes were introduced by standard overlapping PCR mutagenesis using pRep-mCherry as a template with matched forward and reverse mutagenic 3D PCR primers in combination with flanking PCR primers FMDV_4728_45 and FMDV_7200_7180. The second-round PCR product, generated using the products of the first-round PCR in combination with PCR primers FMDV_4728_45 + FMDV_7200_7180, was digested with EcoRI and BamHI and used to replace the equivalent EcoRI-BamHI fragment from pRep-mCherry. The same strategy was used to scramble regions in the 3D coding sequence, using mutagenic PCR primer pairs FMDV_3D_214-221_scrbl_fwd + FMDV_3D_214-221_scrbl_rvs and FMDV_3D_239-246_scrbl_fwd + FMDV_3D_239-246_scrbl_rvs.

Deletion of the S fragment was performed by two-step overlapping PCR mutagenesis with first-round reactions using primer pairs FMDV_Sfrag5′Eagl_rvs + FMDV_JW1_fwd and FMDV_Sfrag5′EagI_fwd + FMDV_Sfrag_3′Eagl_rvs. Products from the first round were used in second-round PCR with primers FMDV_JW1_fwd + FMDV_Sfrag3′EagI_rvs, and the PCR product was digested with NheI + SspI and cloned into NheI + SspI-digested pRep-mCherry to generate pRep-mCherry-Eagl. The S fragment was subsequently removed by digestion of pRep-mCherry-EagI with EagI before religation to generate pRep-mCherry-ΔS.

To introduce the previously published replication-defective CRE point mutation (5) into pRep-mCherry, PCR was used with primers FMDV_CRE_A1G_fwd + FMDV_1331-1311, and the PCR product was digested with AatII and KpnI and ligated into AatII + KpnI-digested pRep-mCherry to generate pRep-mCherry-CRE^A1G^.

Introduction of C-terminal 3D FLAG and hemagglutinin (HA) epitope tags first required introduction of a unique BspEI restriction enzyme site within the 3′ UTR to generate pRep-ptGFP-BseEI. This construct was subsequently used as the template in a two-step PCR, the first-round reactions involving primer pairs 3D_FLAG_fwd or 3D_HA_fwd with FMDV_BspEI_7643_21_rvs and 3D_FLAG_rvs or 3D_HA_rvs with FMDV_6299_6319. The second-round PCR product, generated using the two first-round PCR products with flanking primer pair FMDV_6299 + FMDV_BspEI_7643_21_rvs, was digested with BamHI + BspEI and used to replace the equivalent BamHI + BspEI fragment from pRep-ptGFP-BseEI, to generate pRep-ptGFP-3D^HA^ and pRep-ptGFP-3D^FLAG^. Insertion of 3D point mutations was performed using the strategy described above. To replace the ptGFP reporter gene for Renilla luciferase, the Renilla luciferase reporter gene was amplified by PCR using primers RenLuc_NdeI_fwd and RenLuc_XmaI_rvs and used to replace the ptGFP reporter gene by NdeI + XmaI restriction enzyme digest. Primer sequences are available on request.

### *In vitro* transcription.

*In vitro* transcription reactions were performed as described previously (56). Briefly, 5 μg of replicon plasmid was linearized with AscI (NEB), purified by phenol-chloroform extraction, ethanol precipitated, redissolved in RNase-free water, and used in a 25-μl T7 *in vitro* transcription reaction mixture. Reaction mixtures were incubated at 32°C for 4 h and treated with 1.25 units of RQ1 DNase for 30 min at 37°C, and RNA was recovered using a RNA Clean & Concentrator-25 spin column kit (Zymo Research), in accordance with the manufacturer's instructions. All transcripts were quantified by NanoDrop 1000 (Thermo Scientific), and RNA integrity was assessed by morpholinepropanesulfonic acid (MOPS)-formaldehyde gel electrophoresis before transfection.

### Replication and complementation assays.

BHK-21-based replicon replication assays were performed in 24-well plates with 0.5 μg/cm^2^ of RNA using Lipofectin transfection reagent (Life Technologies) as previously described (56). For complementation assays, BHK-21 cells seeded into 24-well plates were allowed to adhere for 16 h before simultaneous cotransfection with 0.5 μg of both ptGFP and mCherry replicon RNAs using Lipofectin (Life Technologies) according to the manufacturer's instructions. For every experiment, each transfection was performed in duplicate, and experiments were subsequently biologically repeated as indicated. Replicon replication was assessed by live-cell imaging using an IncuCyte Zoom Dual color FLR, an automated phase-contrast and fluorescence microscope within a humidifying incubator. At hourly intervals up to 24 h posttransfection, 9 images of each well were taken and used to enumerate the ptGFP/mCherry-positive cells per well and total ptGFP/mCherry fluorescence intensity per well, using the integrated software. For each experiment, the data were plotted as both positive cell counts per well and total fluorescent intensity. No difference was observed when the data were analyzed as either total cell counts or total fluorescent intensity. Unless otherwise stated, the data shown here represent fluorescence-positive cell counts per well.

For DNA transfection of BSR-T7 cells, 6.25 × 10^4^ cells/cm^2^ seeded into 12-well plates were allowed to adhere for 16 h before transfection with 0.5 μg/cm^2^ of replicon plasmid DNA using Lipofectin reagent (Life Technologies) according to the manufacturer's instructions.

### Isolation of recombinant genomes.

Following cotransfection of BHK-21 cells with mCherry and ptGFP *in vitro*-transcribed replicon RNA, cells were detached by trypsin and washed once in ice-cold phosphate-buffered saline (PBS), and total RNA was extracted using the RNeasy Plus RNA extraction kit (Qiagen) according to the manufacturer's protocol. Total RNA was treated on-column with RQ1-DNase, and total FMDV cDNA was amplified using Superscript II (Life Technologies) according to the manufacturer's protocol. Both mCherry and ptGFP replicon genomes were amplified separately using forward primers specific for either mCherry (mCherry_fwd) or ptGFP (ptGFP_fwd) with FMDV-specific reverse primer (FMDV_7200_7180) with Phusion High-Fidelity DNA polymerase (NEB). PCR products were blunt-end ligated into pCRBlunt. Individual colonies were isolated, and the presence of insertional mutation was determined by DNA sequencing.

### Western blotting.

Immunoblotting was performed as described previously (81) with primary antibodies rabbit anti-3D 397 polyclonal, mouse anti-3A 2C2 monoclonal, and mouse anti-3B 1F8 monoclonal (all generous gifts from Francisco Sobrino, Centro De Biologia Molecular Severo Ochoa, Madrid, Spain), and detection was done with anti-rabbit-horseradish peroxidase (HRP) (Sigma-Aldrich) or anti-mouse-HRP (Sigma-Aldrich) secondary antibodies, as appropriate. A mouse anti-glyceraldehyde-3-phosphate dehydrogenase (anti-GAPDH; Sigma-Aldrich) primary antibody was used as a loading control.

### Immunofluorescence.

BHK-21 cells seeded onto glass coverslips were cotransfected with *in vitro*-produced transcripts, fixed at the indicated time in 4% paraformaldehyde, washed in PBS, and permeabilized in saponin buffer (0.1% saponin, 10% FCS, 0.1% sodium azide) for 1 h at 4°C. Primary antibodies rabbit anti-FLAG (Sigma-Aldrich) and mouse anti-HA (Sigma-Aldrich) were added in saponin buffer for 2 h at room temperature. Secondary antibodies used for detection were anti-rabbit-Alexa Fluor 568 and anti-mouse-Alexa Fluor 647 (Life Technologies), as appropriate. Coverslips were washed three times in saponin buffer between antibody steps. Coverslips were washed a final time in PBS before mounting in ProLong Gold with 4′,6-diamidino-2-phenylindole (DAPI; Life Technologies). Images were captured using a Zeiss LSM-880 inverted confocal microscope.

### Protein purification and crystallization.

The GC216/7AA mutation was introduced into the His-tagged 3D expression clone pET28a-3D (as described in references 31 and 76), using standard molecular cloning techniques. Mutant polymerase was expressed and purified as previously described (31, 76). A last purification step was required for crystallization; a size exclusion on a Superdex 200 16/60 (GE Healthcare) with the protein buffer (500 mM NaCl, 50 mM Tris-HCl [pH 8.0], 8% glycerol, 0.8 mM dithiothreitol [DTT], and 0.8 mM EDTA). The protein was concentrated to 5 mg/ml using an Amicon Ultra concentrator (10.000 MWCO PES; Millipore) previously equilibrated with protein buffer. The protein was aliquoted, frozen, and stored at 193 K. The purity and homogeneity of the protein were analyzed by SDS-PAGE. The oligonucleotide 5′-GCAUGGGCCC (NWG-Biotech) was annealed as described previously (31). 3D was added slowly to the annealed oligonucleotide in the presence of 2 mM MgCl_2_, to reach equimolar proportions. Crystallization was performed by the hanging-drop vapor diffusion method at 293 K in a 24-well Greiner plate. One microliter of protein solution was mixed with 1 μl of precipitant solution; the volume reservoir was 1 ml. The best crystals appeared in 2 or 3 days from a solution containing 30% polyethylene glycol (PEG) 4000, 0.2 M magnesium acetate, 0.1 M MES (morpholineethanesulfonic acid [pH 6.0]), and 4% ϕ-butyrolactone.

### X-ray diffraction analysis.

The 3D-GC216/7AA data sets were collected at the Alba-Cells synchrotron light facility (Barcelona) on a Pilatus 6M Dectris detector at beamline Xaloc using a wavelength (λ) of 0.979 Å. All the data sets were recorded at 100 K. Crystals diffracted to 2.8-Å resolution (Table 1). Four hundred images were collected with an oscillation angle of 0.25° and 0.66 s of exposure time. Diffraction images were processed with XDS (82, 83) and internally scaled with SCALA (CCP4i [84]) (Table 1).

**TABLE 1:** Refinement statistics

Collection and refinement	Value(s)^*c*^
Datum collected	
Resolution (Å)	47.95–2.8 (2.95–2.8)
Space group	P3_2_21
Cell dimensions	
a, b, c (Å)	95.78, 95.78, 100.88
α, β, γ (°)	90, 90, 120
Rmerge (%)	8.3 (76.8)
I/σI	
Completeness (%)	95.3 (93.7)
Multiplicity	2.2 (2.0)
Refinement	
Resolution (Å)	47.95–2.8
No. of reflections (total/unique)	28,367/12,908
Rwork^*a*^/Rfree^*b*^	21.63/28.44
No. of atoms/residues	
Protein	476
Ligand	14 RNA
Water	9H_2_O/1Mg^2+^
B factors (Å^2^)	
Overall	24.002
Protein	23.316
RNA	31.785
Water + ligands	46.102
Rmsd	
Bond lengths (Å)	0.0079
Bond angles (°)	1.2165
Ramachandran plot	
Residues in preferred regions	459/0476 (96.8%)
Residues in allowed regions	14/476 (3%)

a Rwork = ∑hkl ||Fobs(hkl)| − |Fcalc(hkl)||/∑hkl |Fobs(hkl)|, where Fobs and Fcalc are the structure factors, deduced from measured intensities and calculated from the model, respectively.

b Rfree is calculated as for Rwork but for 5% of the total reflections chosen at random and omitted from refinement.

c Values in parentheses correspond to the highest-resolution shell (PDB accession number 5JXS).

### Structure solution and refinement.

The initial maps were obtained after rigid-body fitting of the coordinates of the wild-type 3D, crystallized in the trigonal space group P3_2_21 (PDB 1WNE [31]), after removing the bound RNA using the program Refmac5 (CCP4i [85]) (Table 1). The difference density maps were clear enough to allow retracing of the mutated residues and the RNA template-primer decanucleotide bound to the polymerase central cavity. Several cycles of automatic refinement, performed with Refmac5 (CCP4i [85]) and Phenix software (86) were alternated with manual model rebuilding using Coot (87, 88). The refinement statistics are summarized in Table 1.

### Protein structure accession number.

The crystallographic coordinates determined in this study have been deposited in the Protein Data Bank, with accession number 5JXS.

## RESULTS

### Replication-defective 3D insertional mutations can be recovered by cotransfection with a wild-type replicon.

Due to the limited coding capacity of positive-strand RNA viruses, many viral proteins have multifunctional roles in RNA replication. Several studies have demonstrated that the replication of picornaviruses containing replication-defective mutations in some NS proteins can be recovered by expressing the corresponding wild-type protein in *trans* (i.e., from a separate RNA molecule) (35–40). This is in contrast to certain NS protein functions that are seemingly required in *cis* (i.e., expressed from the same molecule). In this study, we set out to investigate such differential functions of FMDV 3D.

**FIG 1:**
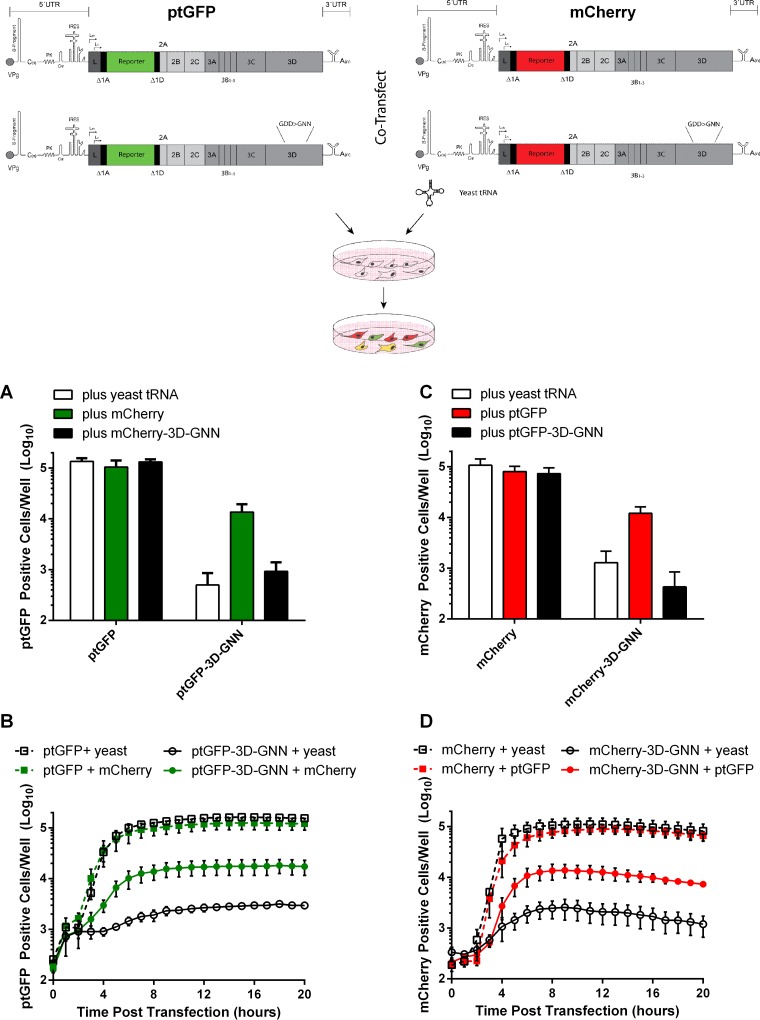
Cartoon of the FMDV replicons used in complementation assays. Schematics of the FMDV subgenomic replicons used in this study. Complementation experiments involved simultaneous cotransfection of BHK-21 cells with equal amounts of replication-defective “mutant” RNA with helper replicon or yeast tRNA as a negative control. Replication of mutant and helper constructs was subsequently monitored by differential expression of fluorescent reporter transgenes encoding ptGFP and mCherry. Expression of ptGFP (A and B) and mCherry (C and D) was monitored hourly over a 24-h period. (A and C) Data are typically represented as mean positive cells per well ± standard deviations (SD) at 8 h posttransfection. (B and D) Time course of fluorescence expression over 20 h from a subset of samples (*n* = 3). The point mutation to the 3D active-site motif C (3D-GNN) has previously been shown to be recovered in *trans* by cotransfection with wild-type replicon.

**FIG 2:**
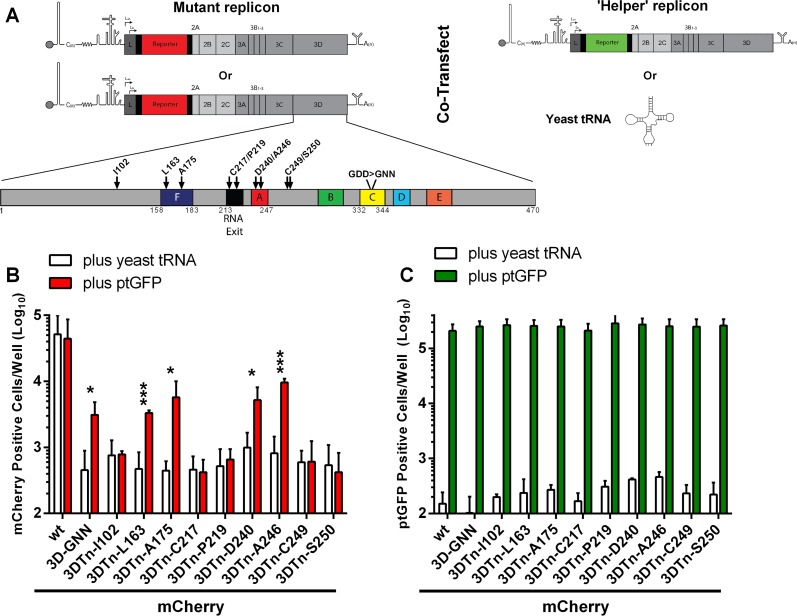
Replication-defective 3D insertions can be recovered using wild-type helper replicon. (A) Cartoon of the FMDV replicon, including a schematic of the 3D sequence with conserved functional regions highlighted. The seven conserved motifs are colored as follows: A, red; B, green; C, yellow; D, light blue; E, orange; and F, dark blue. The region shown to interact with dsRNA is shaded in black. Complementation experiments involved cotransfecting mCherry replicons containing replication-defective 3D insertions, indicated with arrows, with wild-type ptGFP helper replicons or yeast tRNA. BHK-21 cells were seeded into 24-well plates and allowed to adhere for 16 h. Cells were cotransfected with mCherry replicons bearing replication-defective 3D insertions, a motif C active-site 3D mutation (3D-GNN), or a wild-type (wt) mCherry replicon control, together with wild-type ptGFP helper replicon or yeast tRNA as a negative control. Expression of mCherry (B) and ptGFP (C) was monitored hourly over a 24-h period. Data are represented as mean positive cells per well ± SD at 8 h posttransfection. Significance between plus ptGFP and plus yeast tRNA control (*n* = 3): *, *P* < 0.05; **, *P* < 0.01; ***, *P* < 0.001.

We recently reported the use of replication-defective transposon-mediated insertional mutagenesis to reveal replication-defective mutations across the FMDV NS polyprotein (56). In the same report, we demonstrated that replication-defective insertions in the 3A NS protein could be recovered in *trans* by cotransfection with wild-type “helper” replicons in a complementation assay using replicons with different reporters. We also observed that a replication-defective point mutation in motif C of 3D (3D-GNN) could be recovered using a wild-type helper replicon (Fig. 1). We had also identified nine separate replication-defective insertion mutations within 3D (as summarized in Table 2) and therefore first sought to investigate whether such replication-defective 3D insertion mutations could be recovered by cotransfection with helper replicons in complementation experiments. To this end, the nine replication-defective 3D insertion mutations (3DTn-I102 to 3DTn-S250, the name being derived from the amino acid position after which transposon insertion occurred [56]) were introduced into an FMDV replicon in which the structural proteins had been substituted for the reporter protein mCherry (as described in reference 56), thus allowing replication to be monitored via fluorescent transgene expression (81, 89) (Fig. 2A).

**TABLE 2:** Locations of replication-defective 3D insertions^*a*^

Name	Nucleotide insertion site	Nucleotide insertion	Amino acid sequence
3DTn-I102	306	**C**AAGTGCGGCCGCATCA	IYEA**I**KCGRIKGVDG
3DTn-L163	489	**G**AATGCGGCCGCACTGA	CQTF**L**NAAALKDEIR
3DTn-A175	525	**G**GTGCGGCCGCAGCCGG	EKVR**A**GAAAAGKTRI
3DTn-C217	651	**C**AACTGCGGCCGCAGCA	SAVG**C**NCGRSNPDVD
3DTn-P219	657	**C**TGATTGCGGCCGCACT	VGCN**P**DCGRTDVDWQ
3DTn-D240	716	**A**TGCGGCCGCAGTGGAC	VWDV**D**AAAVDYSAFD
3DTn-A246	738	**T**AATGCGGCCGCAGCTA	SAFD**A**NAAAANHCSD
3DTn-C249	747	**T**AGTTGCGGCCGCAGTA	DANH**C**SCGRSSDAMN
3DTn-S250	750	**G**TTGCGGCCGCAGTAGT	ANHC**S**CGRSSDAMNI

a The nucleotide or amino acid after which insertion occurred is shown in bold. The inserted sequence is underlined.

As expected, all nine 3D insertions were replication defective in the context of the mCherry reporter replicon, with mCherry expression approximately equivalent to the replication-defective control construct 3D-GNN (Fig. 2B). After confirming that all nine 3D insertions were replication defective, the ability of these constructs to be recovered was screened by cotransfection with a wild-type helper replicon expressing ptGFP in place of mCherry (as described in reference 56) or yeast tRNA as a negative control for input translation. The use of two different fluorescent reporters allowed for discrimination of replication between the mutant and helper replicons. Alongside, the wild-type mCherry replicon and the mCherry replicon bearing the point mutation to the 3D motif C active site (3D-GNN) (90, 91) were also cotransfected with helper replicon or yeast tRNA. Reporter gene expression was monitored over a 24-h period with an IncuCyte Zoom FLR (as described in Materials and Methods), with data presented at 8 h posttransfection, when reporter gene expression was maximal (Fig. 2B and C).

As described above, the replicon containing the motif C 3D-GNN active-site mutation could be recovered by cotransfection with a wild-type helper replicon (Fig. 2B). With the wild-type mCherry replicon, there was no significant difference in mCherry expression when cotransfected with yeast tRNA control or the wild-type helper replicon, as expected. Four of the replicons bearing replication-defective 3D insertion mutations (3DTn-L163, 3DTn-A175, 3DTn-D240, 3DTn-A246) were recovered by cotransfection with helper replicon, all demonstrating at least a 5-fold, significant increase in replication compared to the yeast tRNA negative control (Fig. 2B). No significant increase in mCherry expression was observed with the remaining five replication-defective replicons containing insertion mutations 3DTn-I102, 3DTn-C217, 3DTn-P219, 3DTn-C249, and 3DTn-S250. There was no significant difference in wild-type ptGFP helper replicon replication when cotransfected with any of the mCherry replicons (Fig. 2C).

The published crystal structures of 3D alone and in complex with RNA (31, 43–46, 92) allowed the locations of the replication-defective insertions to be identified structurally. Insertion mutations 3DTn-L163 and 3DTn-A175 were within the finger domain motif F, involved in interacting with the entry of incoming rNTPs. The two remaining recoverable insertion mutations, 3DTn-D240 and 3DTn-A246, were within the palm motif A, proximal to two catalytic aspartic acid residues (D240 and D245) responsible for nucleotide selection and binding and involved in stabilizing the interaction with the catalytic metal ions.

The five nonrecoverable insertion mutations also located to two distinct sites. Insertion mutations 3DTn-C217 and 3DTn-P219 were localized within the loop region, which, based on the crystal structure of 3D in complex with RNA, is hypothesized to directly participate in the protein-RNA contacts in the double-stranded RNA (dsRNA) exit site. Insertion mutations 3DTn-I102, 3DTn-C249, and 3DTn-S250 were located on the polymerase surface and are predicted to be involved in 3D protein-protein interactions directing the crystal packing in different 3D crystal forms (31, 43–45).

### Recovery of replication-defective 3D mutations is not solely through recombination.

The presence of small replication-defective insertional mutations in 3D acts as a genetic marker allowing the replication-defective genomes to be easily distinguished from the replication-competent “helper” genomes by PCR. Cotransfection of replication-defective with replication-competent genomes might be expected to give rise to replicative recombinants through polymerase strand switching.

To investigate whether we could detect recombinants or wild-type revertants, the mCherry replicons bearing the replication-defective 3D insertional mutations (3DTn-I102 to 3DTn-S250) were cotransfected with the wild-type ptGFP helper construct, the ptGFP-3D-GNN construct, or yeast tRNA controls and RNA extracted at 8 h posttransfection. Both mCherry and ptGFP replicon genomes were amplified separately by reverse transcription-PCR using forward primers specific for mCherry or ptGFP, as appropriate. As anticipated, an mCherry-specific band of the expected size could be observed only when the 3DTn-L163, 3DTn-3DTn-A175, 3DTn-D240, and 3DTn-A246 mutant replicons were cotransfected with wild-type ptGFP helper replicon (data not shown). To isolate recombinants, this PCR product was subcloned into a plasmid vector, and the nonstructural region was sequenced from individual clones.

A total of 20 clones of the 4 recoverable 3D insertions (3DTn-L163, 3DTn-3DTn-A175, 3DTn-D240, and 3DTn-A246) were sequenced. The insertional mutation was clearly identifiable in 14 clones, with 6 clones having lost the insertional mutation, thus having a wild-type sequence (data not shown). No other point mutations were identified across the sequenced region. These data would suggest that the majority of genomes are not recombinants and that the recovery observed is not solely through the effect of recombination. However, recombinant genomes were clearly detected, presumably as a result of polymerase strand switching within mixed replication complexes.

### Point mutations within 3D catalytic motifs can be recovered by cotransfection with wild-type replicon.

The results above showed that approximately one-half of the replication-defective 3D insertions could be recovered by cotransfection with a wild-type helper replicon. The other half were nonrecoverable with a wild-type construct, suggesting differential *trans* (i.e., recoverable with helper replicon) and *cis* (i.e., not recoverable) functions of 3D. However, all of these mutations represent 5 amino acid insertions, which could cause a general disruption to protein folding and stability, as opposed to point mutations designed to abrogate a specific protein function (such as a GNN active-site point mutation). We therefore sought to replicate the phenotypes observed above with the replication-defective 3D insertions with site-directed point mutations. For this, we chose one location that was recovered and one that was nonrecoverable; these were the replication-defective mutations in motif A and the dsRNA exit site, respectively.

**FIG 3:**
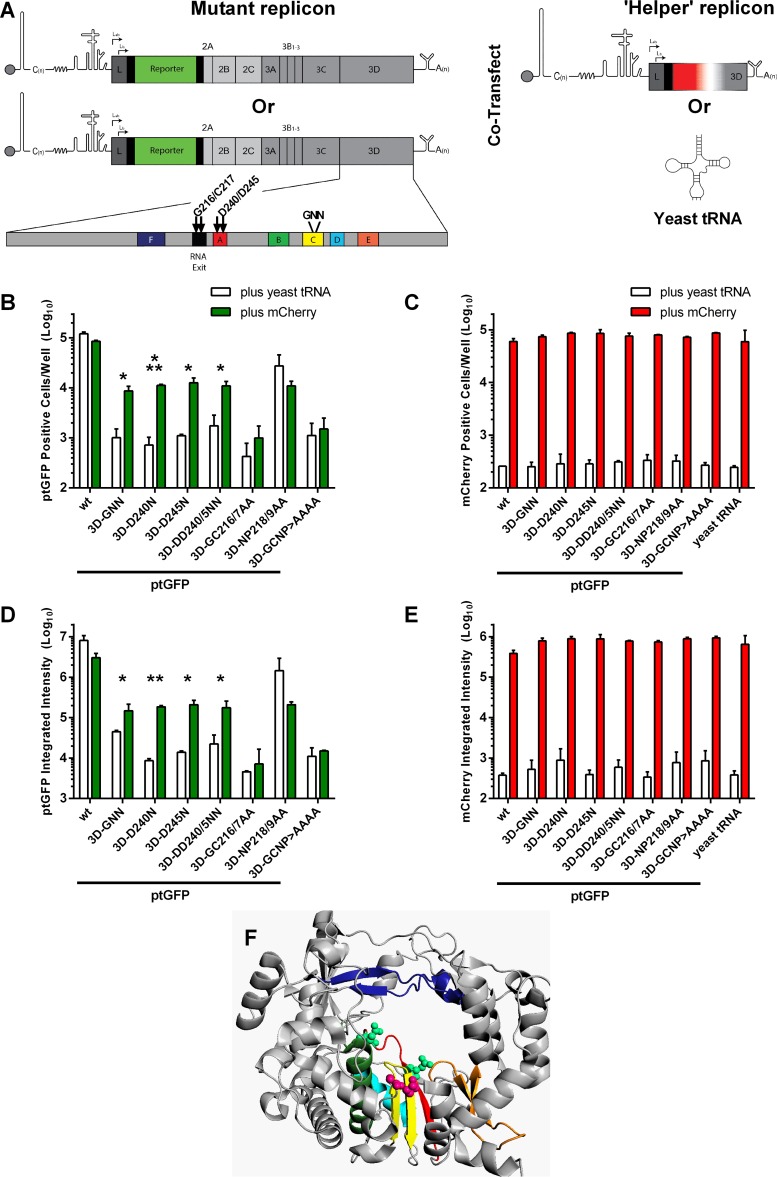
Replication-defective mutations in 3D motifs A and C can be recovered using wild-type helper replicon. (A) Cartoon of the FMDV replicon bearing replication-defective polymerase point mutations and a simplified version of the mCherry helper replicon (see Fig. 1 for full schematic). The schematic of the 3D polymerase gene is shown with the conserved motifs A to F highlighted. Arrows indicate the locations of mutated residues. Complementation assays involved cotransfection of ptGFP replicons bearing 3D point mutations with wild-type mCherry helper replicon or yeast tRNA. BHK-21 cells seeded into 24-well plates were cotransfected with ptGFP replicons harboring replication-defective 3D point mutations or a wild-type control, together with wild-type mCherry helper replicon or yeast tRNA as a negative control. Expression of ptGFP (B and D) and mCherry (C and E) were monitored hourly over a 24-h period. Data show mean positive cells per well ± SD (B and C) or mean total fluorescent intensity per well ± SD (D and E) at 8 h posttransfection. Significance between plus mCherry and plus yeast tRNA control (*n* = 2): *, *P* < 0.05; **, *P* < 0.01; ***, *P* < 0.001. (F) Diagrammatic representation of the 3D crystal structure showing the position of recoverable and nonrecoverable replication-defective point mutations as light green and hot pink spheres, respectively. Motif C containing the active-site GDD motif is colored yellow. Motifs A and F are colored red and dark blue, respectively.

Replication-defective insertions within motif A, characterized by insertional mutations 3DTn-D240 and 3DTn-A246, were located adjacent to either of the catalytic aspartic acid residues D240 and D245, shown in crystal structures to interact with the catalytic metal ion. We therefore hypothesized that these insertions abrogated the function of these aspartic acid residues, and to investigate this, we generated three new ptGFP replicon constructs bearing either a D240N or D245N point mutation or a DD240/5NN double point mutation. The two replication-defective nucleotide insertion mutations located in the dsRNA exit site, 3DTn-C217 and 3DTn-P219, were situated in close proximity to residues G216 and P219, which are thought to interact with RNA in the dsRNA exit site (31). However, it was difficult to infer from the crystal structures which of these residues are critical to 3D function. Therefore, to identify replication-defective point mutations in this region, we undertook alanine scanning mutagenesis across the residues G216 to P219, to generate three new ptGFP replicon constructs containing either a 3D-GC216/7AA or 3D-NP218/9AA double point mutation or a 3D-GCNP216-219AAAA quadruple mutation (Fig. 3A).

The ability of these six new ptGFP constructs to replicate was assessed in BHK-21 cells along with a wild-type positive control and polymerase active-site 3D-GNN mutation negative control (Fig. 3B). As expected, both 3D-D240N and 3D-D245N mutations and the combined 3D-DD240/5NN double mutation within motif A completely prevented replication (Fig. 3B). Likewise, the dsRNA exit site mutations 3D-GCNP216-9AAAA and 3D-GC216/7AA were both replication defective, with ptGFP expression equivalent to the 3D-GNN motif C knockout control (Fig. 3B). In contrast, the 3D-NP218/9AA mutation in the dsRNA exit site demonstrated robust replication, with only a 2- to 3-fold decrease in ptGFP expression compared to the wild-type construct (Fig. 3B). From this we concluded that disruption of residues G216 and C217 was responsible for the replication-defective phenotype in the dsRNA exit site.

Next, we determined whether these replication-defective point mutations in 3D motif A or the dsRNA exit site could be recovered in a complementation assay (as described in the legend to Fig. 3A). For pragmatic reasons from this point on, all complementation assays were performed with mutant ptGFP replicon and helper replicons expressing mCherry. Therefore, the ptGFP replicons bearing mutations 3D-D240N, 3D-D245N, 3D-DD240/5NN, 3D-GC216/7AA, 3D-NP218/9AA, or 3D-GCNP216-9AAAA were cotransfected with the wild-type mCherry helper replicon or yeast tRNA as a negative control, and replication was monitored by transgene expression. The wild-type and 3D-GNN ptGFP replicons were also included as controls (Fig. 3B and C).

The three motif A mutations (3D-D240N, 3D-D245N, and 3D-DD240/5NN) were significantly recovered by cotransfection with a wild-type helper mCherry replicon (Fig. 3B). In contrast, neither of the dsRNA exit site point mutations (3D-GC216/7AA or 3D-GCNP216-9AAAA) was recovered, with no significant increase in ptGFP expression when cotransfected with helper construct compared to yeast tRNA control (Fig. 3B). There was no significant difference in ptGFP expression with the 3D-NP218/9AA construct when cotransfected with a helper construct compared to the yeast tRNA negative control. Again, there was no significant difference in replication of the helper construct when cotransfected with the wild-type or any of the replication-defective 3D replicons, compared to the yeast tRNA control (Fig. 3C), suggesting no dominant negative effects on replication with any of the 3D mutations. The same results were observed when analyzing expression as either total ptGFP and mCherry fluorescent intensity or as total fluorescent cell counts per well; for completeness, this is shown in Fig. 3D and E. Thus, the results with 3D point mutations correspond to those observed above with replication-defective 3D insertion mutations (Fig. 2), with motif A mutations significantly recovered while dsRNA exit site mutations were unrecoverable (Fig. 3F). Such observations implicate different *trans* and *cis* functions of 3D that are essential for viral replication.

### Replication-defective 3D point mutations do not change RNA structure.

**FIG 4:**
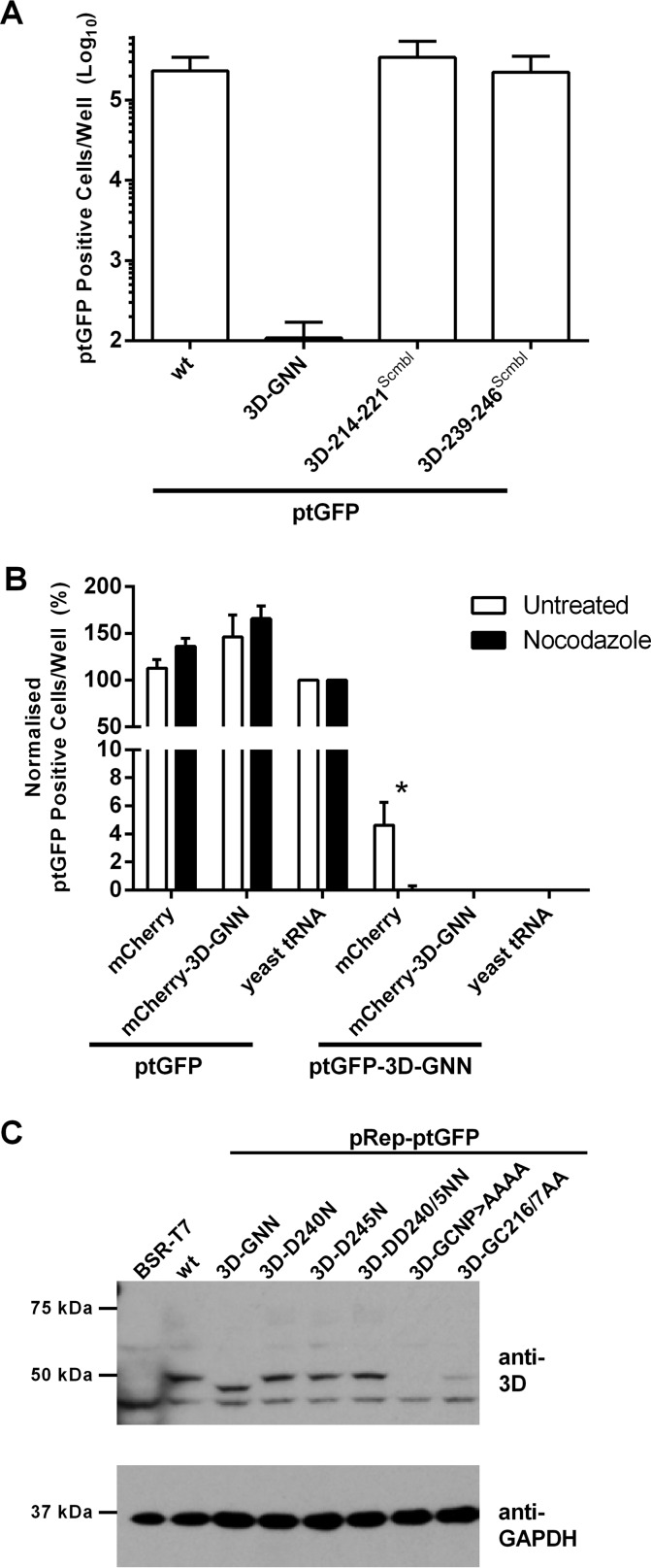
Replication-defective 3D mutations do not affect RNA structure or 3D polyprotein processing. (A) Replicon RNAs containing codon-modified 3D were transfected into BHK-21 cells along with control replicons, and ptGFP expression was monitored hourly over 24 h. Data show mean numbers ± SD of ptGFP-positive cells per well at 8 h posttransfection (*n* = 2). (B) Effect of nocodazole on complementation. BHK-21 cells treated with 5 μM nocodazole or left untreated (see Materials and Methods for details) were cotransfected with wild-type or 3D-GNN ptGFP replicon RNA together with helper mCherry replicon or yeast tRNA negative control. ptGFP expression was monitored for 24 h. Data show normalized numbers of ptGFP-positive cells per well ± standard errors of the means (SEM) at 8 h posttransfection. Significance between results for untreated samples and samples treated with 5 μM nocodazole (*n* = 3) was determined: *, *P* < 0.05. (C) Western blot analysis of replicon DNA constructs transfected into BSR-T7 cells. Cells were seeded into 12-well plates, allowed to adhere for 16 h, and transfected with ptGFP replicon DNA constructs as indicated, and protein was harvested at 12 h posttransfection. Lysates were analyzed by Western blotting for 3D and GAPDH (glyceraldehyde-3-phosphate dehydrogenase) expression.

Previous studies have described RNA structural elements within the 3D coding region of some picornaviruses, which are hypothesized to have an essential role in viral RNA replication (93). It was possible that the mutations introduced within the 3D motif A (3D-DD240/5NN) or the dsRNA exit site (3D-GC216/7AA) inhibited replication by disruption of specific local RNA sequence, as opposed to disruption of protein function. To investigate this possibility, the 3D codon usage spanning the 8 residues comprising the whole of motif A (residues V239 to A246) or the whole dsRNA exit site (residues A214 to V221) were changed in the ptGFP replicon to maintain the amino acid sequence while introducing the maximum number of changes to the nucleotide sequence. These two new “scrambled” replicons, 3D-214-221^Scmbl^ and 3D-239-246^Scmbl^, therefore maintained the amino acid sequence of 3D, but with a different RNA sequence. The replication of both scrambled replicon constructs was assessed in BHK-21 cells along with wild-type ptGFP positive control and the motif C polymerase knockout negative control (3D-GNN) (Fig. 4A). Both replicons with a scrambled nucleotide sequence across either motif A or the dsRNA exit site replicated in a fashion similar to that of the wild-type construct. These data suggest that the replication-defective mutations introduced into either motif A or the dsRNA exit site were due to changes in the 3D protein sequence and were not a result of local change in the RNA sequence.

### Nocodazole inhibition of complementation.

Studies with PV have indicated that transient cell chilling followed by the addition of the drug nocodazole blocked complementation by the depolymerization of microtubules and prevention of coalescence of replication complexes (94, 95). To investigate the influence of nocodazole on FMDV complementation, the ptGFP-3D-GNN replicon was cotransfected with the wild-type mCherry helper construct, the mCherry-3D-GNN replicon, or yeast tRNA following treatment with nocodazole. The wild-type ptGFP replicon was included as an internal control and recovery of replication assessed via fluorescent protein expression over time, with data showing maximal ptGFP expression at 8 h posttransfection (Fig. 4B).

Contrary to data published for PV, treatment with 5 μM nocodazole resulted in approximately 30% reduction in replicon replication (data not shown). Despite this reduction, normalization of the data revealed that treatment with nocodazole significantly inhibited recovery of the 3D-GNN construct by wild-type helper replicon (Fig. 4B). These data suggest that recovery of replication-defective 3D mutations involves a process that requires mixing of replication complexes.

### Replication-defective 3D point mutations do not affect P3 processing.

Having confirmed that the replication-defective changes to 3D were a result of protein modification and not local RNA changes, we sought to identify whether such mutations affected 3D processing or stability. Directly transfecting replicon DNA into BSR-T7 cells (BHK-21 cells stably expressing T7 polymerase) allows for continuous replicon transcription and translation in the absence of replication, thus allowing for the detection of nonstructural proteins from replication-defective constructs. BSR-T7 cells were therefore transfected with replicon construct DNA containing the 3D motif A and dsRNA exit site mutations along with wild-type and 3D-GNN controls. Western blot analysis revealed no notable difference in 3D precursors between the wild-type and mutant constructs (Fig. 4C). There was also no notable difference in 3D expression levels between the wild-type constructs and constructs carrying the motif A (3D-D240N, 3D-D245N, 3D-DD240/5NN) or motif C (3D-GNN) mutations. In contrast, there was reduced 3D expression from both the dsRNA exit site mutants (3D-GC216/7AA and 3D-GCNP216-9AAAA), in particular the quadruple mutant, which showed very little expression of 3D, suggesting that mutations in this region have detrimental effects on protein stability. However, there was a smaller reduction in 3D expression from the double mutant 3D-GC216/7AA. For this reason we chose this double mutation, in addition to the 3D-DD240/5NN double point mutation in motif A and 3D-GNN mutation in motif C, for further study.

### Replication-defective 3D colocalizes with wild-type 3D in replicon-cotransfected cells.

**FIG 5:**
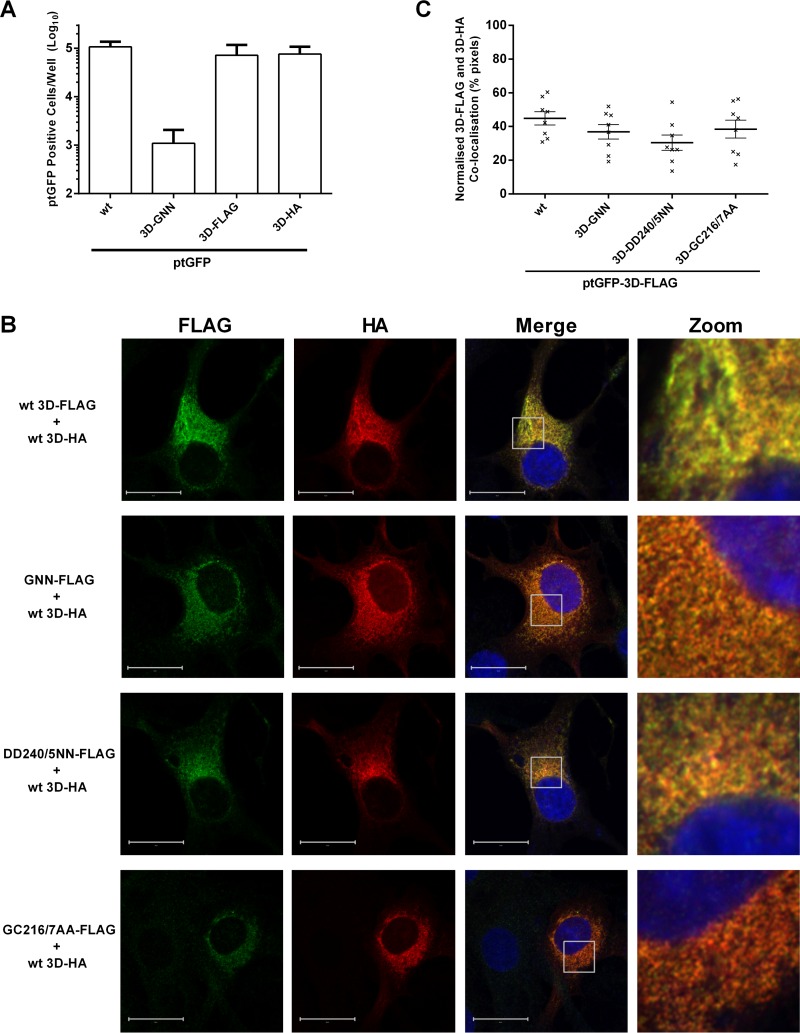
Wild-type helper 3D colocalizes with replication-defective mutant 3D. (A) Replicon RNAs containing C-terminal epitope labels were transfected into BHK-21 cells, and ptGFP expression was monitored hourly over a 24-h period. Data show mean numbers ± SD of ptGFP-positive cells per well at 8 h posttransfection (*n* = 2). (B) BHK-21 cells were cotransfected with FLAG-tagged mutant 3D replicons and HA-tagged 3D helper replicons, fixed at 4 h posttransfection, and labeled for FLAG-Alexa Fluor 568 (pseudocolored green) and HA-Alexa Fluor 647 (pseudocolored red) as described in Materials and Methods. Cell nuclei were counterstained with DAPI (blue). Images were captured on a Zeiss LSM-880 confocal microscope with Airyscan. Zoomed images represent the boxed areas on the merged images (bar, 20 μM). (C) Quantification of FLAG versus HA colocalization by confocal immunofluorescence. Fluorescent intensity of 3D-FLAG (green) versus 3D-HA (red) was plotted as two-dimensional (2D) scatterplots, and the number of costained pixels was determined by a 4-area gating set using controls. Data points show the percentages of coexpressing pixels from 8 randomly selected cells after cotransfection. Horizontal lines represents mean values ± SEM.

It was anticipated that cotransfection of mutant and helper replicons would result in colocalization of both mutant and helper 3D within the same cellular compartment. Investigation of this required differential labeling of the two subsets of 3D molecules. For this purpose, 3D was modified to contain either a C-terminal FLAG or HA epitope tag within the context of the replicon expressing the ptGFP reporter, allowing the tagged molecules to be detected by indirect immunofluorescence using commercially available antibodies. *In vitro*-synthesized RNA from these new epitope-labeled constructs was transfected into BHK-21 cells along with appropriate wild-type positive and polymerase knockout (3D-GNN) negative controls, and ptGFP expression was monitored over time (Fig. 5A). Both FLAG- and HA-labeled ptGFP replicons yielded ptGFP expression indistinguishable from that of the wild-type ptGFP construct (Fig. 5A), showing that both C-terminal epitope tags were well tolerated and had no detrimental effect on RNA replication.

To check that the replication-defective polymerase mutations did not result in protein mistargeting, the C-terminal FLAG tag was combined with the 3D-DD240/5NN, 3D-GC216/7AA, or 3D-GNN mutations, and RNA was generated and transfected along with wild-type ptGFP-3D-FLAG control into BHK-21 cells. Cells were fixed at 4 h posttransfection, labeled for FLAG-tagged 3D, and analyzed by immunofluorescence (data not shown). There was no appreciable difference in the cellular distribution of mutant and wild-type polymerases, all of which showed a diffuse punctate pattern of staining scattered throughout the cytoplasm (data not shown), albeit with the wild-type replicon displaying greater 3D expression, as would be expected.

The ability to tag 3D with two alternative epitopes (i.e., FLAG or HA) allowed us to study the localization of mutant and helper 3D in replicon-cotransfected cells. Helper constructs were generated in which the mCherry fluorescent reporter was replaced with Renilla luciferase and the 3D modified to contain a C-terminal HA tag to permit dual-color immunofluorescence analysis. The new luciferase-expressing helper constructs were cotransfected into BHK-21 cells along with the ptGFP replicons carrying FLAG-labeled polymerase mutations (3D-GNN, 3D-DD240/5NN, 3D-GC216/7AA) or wild-type control, and cells were fixed at 4 h posttransfection and costained for FLAG and HA expression (Fig. 5B and C). In all cases, colocalization was clearly detected between the wild-type HA-labeled and mutant FLAG-labeled polymerases, indicative of mixed replication complexes. These data provide further evidence that all the replication-defective mutant 3D polymerases can successfully target the presumed site of viral RNA replication when supplied with a wild-type replicon and that recovery of replication occurs through the mixing of replication proteins.

### Removal of 5′-UTR RNA elements from the helper replicon improves recovery of replication-defective 3D mutations.

**FIG 6:**
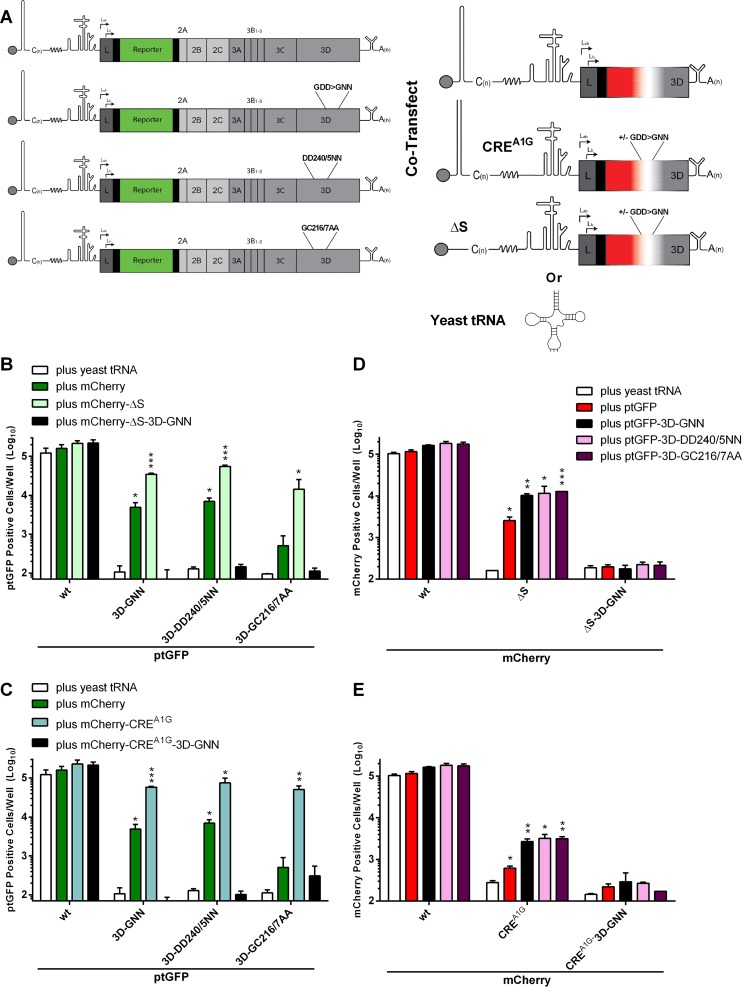
Replication-defective mutations to the 5′ UTR improve recovery of 3D mutations. (A) Schematic of the mutant ptGFP FMDV replicon genome and a simplified version of the mCherry genomes used in the reciprocal complementation assay. BHK-21 cells seeded into 24-well plates were cotransfected with replication-defective 3D mutant ptGFP replicons or wild-type control, together with mCherry replicons containing either an entire S-fragment deletion (ΔS) (B and D) or a replication-defective CRE mutation (CRE^A1G^) (C and E). Wild-type mCherry helper replicon and yeast tRNA cotransfections were included as controls. Expression of ptGFP (B and C) and mCherry (D and E) was monitored hourly over a 24-h period. Data represent mean positive cells per well ± SD at 8 h posttransfection. Significance of differences between treatment and yeast tRNA negative control (*n* = 2): *, *P* < 0.05; **, *P* < 0.01; ***, *P* < 0.001.

There is evidence to suggest that NS proteins of FMDV, either alone or as uncleaved precursor(s), interact with both the CRE and a 5′ RNA structure known as the S fragment (38, 66, 96). We hypothesized that such structured RNA elements in the helper constructs may sequester 3D and/or its precursors in *cis* and so reduce its availability to recover replication-defective replicons. To test this hypothesis, a new set of replication-defective mCherry constructs were generated containing either a previously described functionally inactivating A>G point mutation in the CRE (mCherry-CRE^A1G^) (5) or a complete deletion of the S fragment (mCherry-ΔS). Both of these mutations render the replicon replication defective (data not shown). In addition, two further replicons were built by combining the replication-defective 3D-GNN mutation with either the CRE^A1G^ or ΔS mutations to generate mCherry-CRE^A1G^-3D-GNN and mCherry-ΔS-3D-GNN, respectively. These four new replicons (with functional or nonfunctional 3D) were assayed for their ability to recover replication of the 3D-DD240/5NN, 3D-GC216/7AA, and 3D-GNN mutant constructs, in reciprocal complementation experiments (56) (Fig. 6A to E).

As observed previously, both 3D-GNN and 3D-DD240/5NN mutations were significantly recovered by cotransfection with the wild-type construct. However, recovery was enhanced upon cotransfection with either replication-defective mCherry-ΔS or mCherry-CRE^A1G^ replicons. Consistent with our earlier results, there was no significant increase in ptGFP expression when the 3D-GC216/7AA was cotransfected with a wild-type replicon. However, this mutation showed a >100-fold statistically significant increase in ptGFP expression upon cotransfection with either the mCherry-ΔS (Fig. 6B) or mCherry-CRE^A1G^ replicons (Fig. 6C). No recovery was observed with either of the constructs carrying the replication-defective 3D-GNN mutation, as expected. As before, there was no significant difference in replication of the wild-type mCherry construct when cotransfected with any of the replication-defective 3D replicons. In contrast, the replication of both mCherry-ΔS (Fig. 6D) and mCherry-CRE^A1G^ replicons (Fig. 6E) was significantly recovered upon cotransfection with any of the mutant 3D replicons. Furthermore, ptGFP-3D-GNN, ptGFP-3D-DD240/5NN, and ptGFP-3D-GC216/7AA were each able to recover mCherry-ΔS and mCherry-CRE^A1G^ replication to approximately equal levels.

### Three-dimensional structure of the 3D-GC216/7AA mutant polymerase.

To gather further information on how the GC216/7AA mutation may alter 3D-RNA interactions, the protein was produced as a His-tagged fusion in Escherichia coli as previously described (31, 76) and prepared for crystallization in complex with a short RNA oligomer.

**FIG 7:**
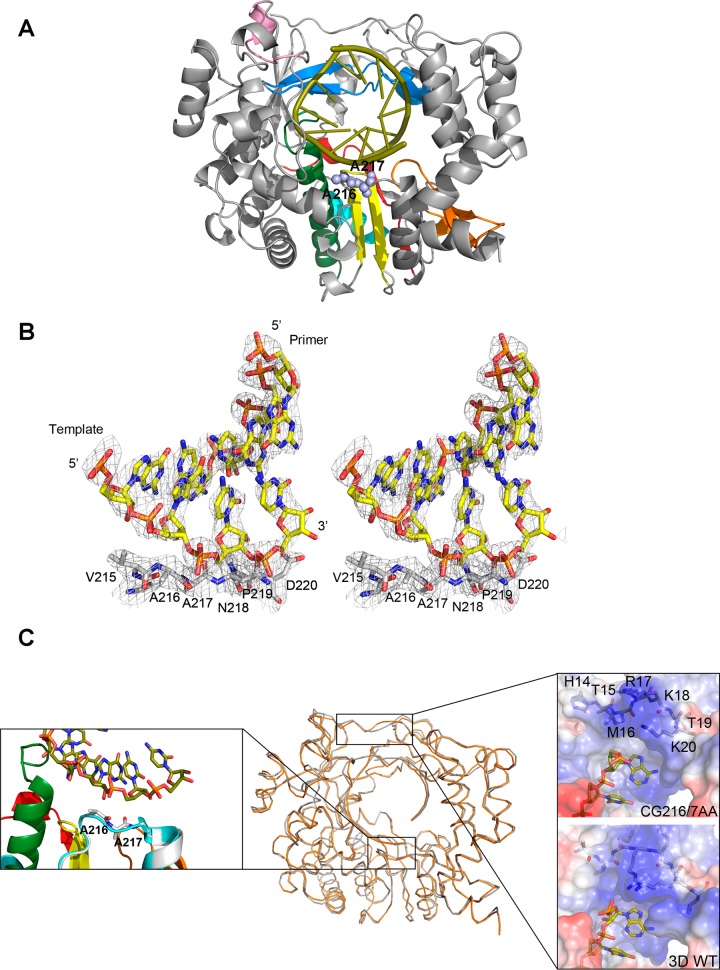
Structure of the 3D-GC216/7AA mutant polymerase in complex with RNA. (A) Front view of a ribbon diagram of the 3D protein (gray) with the substituted amino acids A216 and A217 shown as light blue spheres. The seven conserved structure-sequence motifs are colored as follows: A, red; B, green; C, yellow; D, light blue; E, orange; F, blue; G, pink. (B) Electron density map around residues A216 and A217. Stereoview of a weighted 2F_o_-Fc Fourier map, contoured at 1.5 σ, with the model placed inside (sticks colored in atom type code). (C) Superimposition of the wild-type (orange) and mutant (gray) 3D RNA structures. The regions showing the largest variations are highlighted as close-up insets: the mutated loop (left panel) and the entry of the template channel (right panel).

The X-ray structure of the 3D-GC216/7AA polymerase, in complex with the RNA template/primer 5′-GCAUGGGCCC, was solved at 2.8-Å resolution (Table 1 and Fig. 7A). The initial difference maps allowed unequivocal tracing of the substituted (GC216/7AA) and surrounding (from V215 to D220) residues that were omitted from the initial model to eliminate model bias (Fig. 7A). These maps also indicated the presence of the duplex portion of the template-primer in the central cavity of the enzyme and the AU nucleotides, belonging to the template 5′ overhang region, bound to the template channel (Fig. 7A and B).

Structural comparisons showed that the 3D-GC216/7AA mutant polymerase is almost identical to the wild-type 3D. The superimpositions of all 476 amino acid residues of the 3D-GC216/7AA-RNA complex onto the wild-type 3D-RNA structure (PDB 1WNE) yielded root mean square deviation (rmsd) values of only 0.51 Å. Structural comparisons also showed a subtle movement of the loop containing the mutated residues GC216/7AA, which was displaced by ∼0.74 Å toward the RNA primer (Fig. 7C). However, this small displacement does not alter the 3D-RNA interactions at the dsRNA exit site. The main differences lie in the template entry channel, in particular in the M16-R17 segment, located distal from the mutated region (Fig. 7C). Interestingly, all the structures of the wild-type FMDV 3D catalytic complexes analyzed to date show that the basic side chain of R17 is involved in a different interaction with the template nucleotide at the position t + 2 (44, 45, 97). In these complexes, the t + 2 nucleotide points toward the active-site cavity, stacked with the t + 1 nucleotide that is located in the opening of the central cavity (Fig. 7C, bottom right inset). In contrast, in the GC216/7AA structure, the main and side chains of residues M16 and R17 appeared reoriented, with the R17 side chain pointing to the polymerase interior, interacting with residues N41 and Y285. A similar reorganization of the template channel entry has been previously shown in other FMDV 3D mutants presenting alterations in RNA binding affinity and incoming nucleotide incorporation (46, 98). This rearrangement results in the formation of distinct pockets where the t + 2 nucleotide binds (Fig. 7C, top right inset). These structural data would imply an RNA binding deficiency of this mutant polymerase consistent with the above-described data.

### Expression of 3D alone is not sufficient for recovery of 3D mutations.

**FIG 8:**
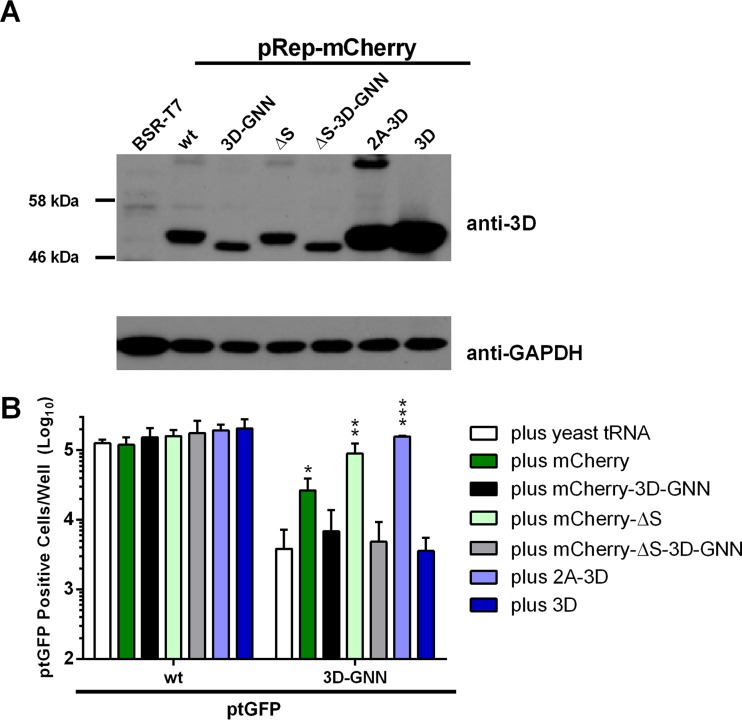
Expression of 3D alone is not sufficient for recovery of 3D mutations. (A) Western blot analysis of helper construct DNA transfected into BSR-T7 cells. Cells were seeded into 12-well plates, allowed to adhere for 16 h, and transfected with DNA constructs as indicated; protein was harvested at 12 h posttransfection. Lysates were analyzed by Western blotting for 3D and GAPDH expression. (B) For reciprocal complementation, BHK-21 cells seeded into 24-well plates were cotransfected with ptGFP replicons bearing the 3D-GNN replication-defective mutation or wild-type control, together with RNA transcripts expressing 3D alone or the entire 2A-3D polyprotein. Cotransfections were also performed with wild-type mCherry or mCherry-ΔS replicon transcripts and the equivalent 3D-GNN mutant constructs. Expression of ptGFP was monitored hourly over a 24-h period. Data represent mean positive cells per well ± SD at 8 h posttransfection. Significance compared to yeast tRNA control (*n* = 3): *, *P* < 0.05; **, *P* < 0.01; ***, *P* < 0.001.

Mature 3D is produced via a series of polyprotein intermediate or precursor proteins. It is generally believed that these precursors provide additional functionality in genome replication. To investigate whether it was possible to recover replication-defective 3D mutations by expression of 3D alone, RNA constructs that express either 3D alone or the entire 2A-3D polyprotein directly from the FMDV IRES were generated. We first verified protein expression by transfection of these two expression constructs into BSR-T7 cells. As controls, BSR-T7 cells were also transfected with wild-type mCherry and mCherry-ΔS replicon DNA (Fig. 8A). As expected, expression of 3D could clearly be detected in all cases.

To assess recovery of the ptGFP-3D-GNN replicon, T7 polymerase *in vitro* RNA transcripts were generated from the 2A-3D and 3D expression constructs, along with controls, and cotransfected into BHK-21 cells as previously described. Cotransfections were also performed with the wild-type ptGFP replicon to check for dominant negative effects (Fig. 8B).

As anticipated, replication of the ptGFP-3D-GNN replicon was recovered by cotransfection with the wild-type helper replicon. This recovery was again improved by cotransfection with the replication-defective mCherry-ΔS transcript. Expression of the full-length 2A-3D polyprotein directly from the FMDV IRES (plus 2A-3D) significantly recovered replication of the 3D-GNN replication-defective replicon, demonstrating an approximately 100-fold increase in ptGFP expression. In contrast, no recovery was observed when expressing 3D alone (plus 3D), compared to the yeast tRNA control (Fig. 8B). Thus, it appears that recovery of 3D mutations requires at least one 3D-containing higher-order precursor.

## DISCUSSION

The ability of some components of picornaviral replication to be supplied in *trans* has been well documented, including the NS proteins 2B, 3A, and 3D and RNA elements such as the CRE and the IRES (36, 39, 40, 54–57, 99, 100). However, it has not always been clear why certain protein or RNA functions can be successfully supplied in *trans* while others cannot. We have recently reported the use of random transposon-mediated mutagenesis of an FMDV replicon to identify sites across the NS polyprotein at which the insertion of foreign sequences abrogates replication (56). In the same report we demonstrated that replication of such replication-defective insertion mutants in the 3A protein could be recovered by cotransfection with a wild-type helper replicon.

In this study, we have extended these observations to investigate replication-defective insertion sites in the viral RdRp gene encoding 3D. In cotransfection complementation experiments, approximately one-half of the replication-defective insertion mutants were significantly recovered in *trans* by a wild-type helper replicon. The insertions in these recoverable mutants were located within catalytic motifs of the polymerase, in particular, motifs A, C, and F. In contrast, insertions in nonrecoverable mutants were located within regions hypothesized to be responsible for protein-protein and/or protein-RNA interactions. Using this information, we designed specific point mutations within the polymerase catalytic domain motif A, which were also significantly recovered in *trans* using a wild-type replicon, and within the putative dsRNA exit site, which were not recoverable with wild-type helper replicon. Such mutations did not detectably alter the processing or cellular localization of 3D. However, structural studies implicated altered RNA binding affinity as being responsible for the biochemical lesion in the 3D protein bearing mutations at the RNA exit site. None of the 3D mutations tested here showed dominant negative effects on the replication of any helper construct. Together, our data imply a novel nonenzymatic, *cis*-acting role of 3D in FMDV replication, which is distinct from the polymerization functions, which can be efficiently supplied in *trans*. We hypothesized that 3D interacts in a *cis*-preferential manner with structured elements within the 5′ UTR and that removal of such elements from the helper replicon would aid recovery of replication-defective 3D mutations by increasing the availability of 3D to function in *trans*. In accordance with this hypothesis, removal of 5′-UTR RNA elements allowed for recovery of *cis*-acting 3D mutations.

**FIG 9:**
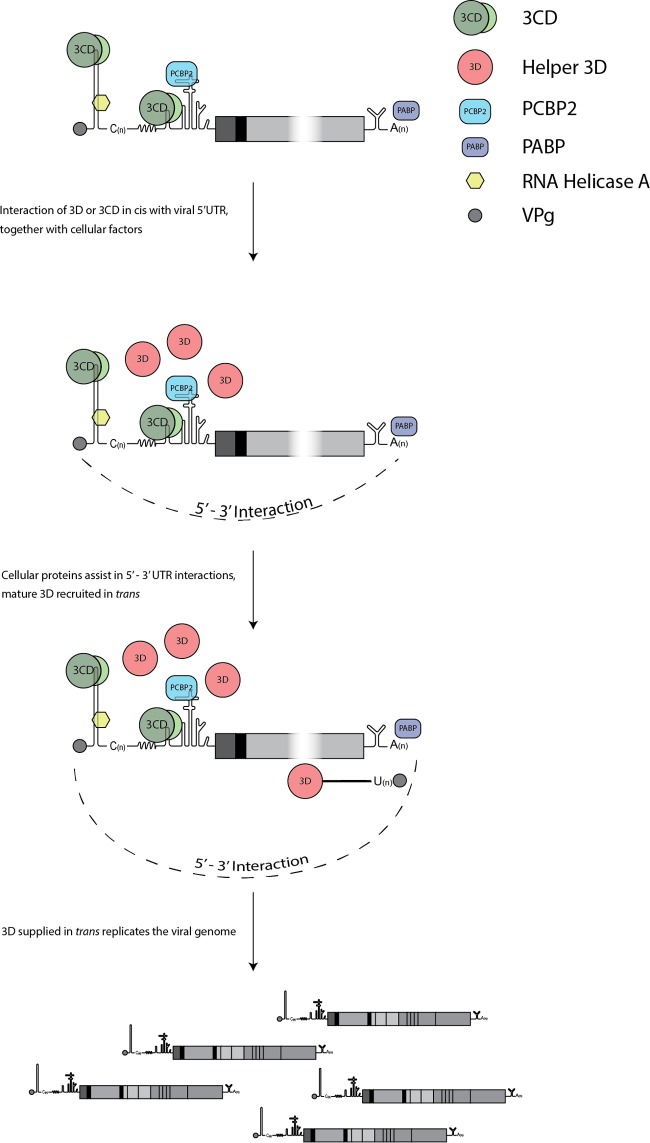
Model of the *cis* and *trans* functions of 3D. 3D, either alone or as part of a larger precursor, interacts in *cis* with structural element within the 5′ UTR. In contrast, the polymerase functions of 3D can be readily supplied in *trans*. Cellular proteins are recruited at both the 5′ and 3′ UTRs. In addition, 5′-3′ RNA interactions are likely to be involved in the formation of an essential ribonucleoprotein complex.

Models for picornavirus genome replication derived from work with PV require a noncovalent interaction between the 5′ and 3′ UTRs involving host cell and viral proteins in a ribonucleoprotein complex to facilitate efficient translation and/or replication (37, 58, 59, 64, 65, 68–73). The 5′ end of the PV genome appears to be required in *cis* for efficient 3D-mediated VPg uridylylation, a process that is stimulated in the presence of 3C or particularly 3CD (37, 49, 52, 53). In addition, it has been observed that 3CD and 3C bind to elements within the 5′ UTRs of PV and FMDV, respectively (59, 66). Biochemically, 3D has been shown to possess no polymerase activity in its precursor form(s), suggesting that processing of 3D from its precursor is required in the replication complex to facilitate VPg uridylylation and genome replication (60–62). Combining these observation with the findings presented in this study, it is tempting to speculate that 3D either alone or as part of a larger precursor, such as P3, binds preferentially in *cis* to the positive-sense RNA to help form the viral ribonucleoprotein complex and facilitate the transition from a translational to a replicating form. Subsequent recruitment of additional molecules of 3D that are readily supplied in *trans* would allow uridylylation of VPg and replication of the genome (Fig. 9). Such a model would support the idea of the involvement of differential 3D protein-protein interactions and higher-order 3D multimers as suggested by genetic and structural data and by recent work on PV in cell-free systems (74–79). Similar genetic complementation studies with other positive-sense RNA viruses such as hepatitis C virus (HCV) have identified essential *cis*-acting functions of viral nonstructural proteins, including the HCV RdRp NS5B (101). Recent work by Kazakov et al. demonstrated that although NS5B polymerization activities could be supplied in *trans*, expression was also required in *cis*, indicative of a *cis*-acting role in replication complex assembly, distinct from the enzymatic activity. Such observations are consistent with the findings presented in this study and could imply a similar *cis*-acting role of RNA polymerases across all positive-sense RNA viruses (101).

The data presented here suggest that recovery of replication-defective mutations occurs through mixing of replication complexes and not through nonreplicative (i.e., homologous) recombination. The majority of replication-defective genomes amplified by reverse transcription-PCR still maintained the mutagenic insertion. However, a small number of recombinant genomes were detected, presumably as a result of polymerase strand switching during RNA replication. This finding, together with our other observations, suggests that recovery occurs through the mixing of replication complexes or sharing of viral NS proteins, and it is therefore likely that genome recombination in our system is a downstream process following from successful complementation. Despite the generally good levels of recovery observed with 3D motif A and motif C mutations, ptGFP expression never achieved the levels observed with the wild-type construct. However, it is important to keep in mind that recovery of replication-defective 3D mutations relies on simultaneous cotransfection of the replication-defective construct with the helper construct. Therefore, the imperfect recovery observed in our system is likely to be largely limited by the intrinsic efficiency of RNA cotransfection, although other possibilities, such as the efficiency of delivering 3D molecules in *trans* between replication complexes, could also play a role. Further studies employing infectious virus as a helper in complementation experiments are therefore warranted to differentiate between these two possibilities.

Of note, our initial insertional mutagenic screening (Fig. 2) also identified replication-defective nonrescuable insertions in the regions predicted to form putative protein-protein interactions (3DTn-I102, 3DTn-C249, and 3DTn-S250). Such observations would be consistent with both our data and previous models, whereby 3D/3CD protein-protein interactions and higher-order 3D multimers are proposed to be essential for ribonucleoprotein and replication complex formation (58, 59, 65, 75–79, 102). Further studies are ongoing to investigate the *cis* versus *trans* viral and host cell protein-protein interactions generated in the formation of the replication complex and how 3D/3CD molecular protein interactions contribute in *cis* as part of a ribonucleoprotein complex and the transition from translation to genome replication.

In summary, we have identified novel *cis* and *trans* functions of the FMDV 3D RNA-dependent RNA polymerase that are essential for viral RNA replication. This includes a previously undescribed nonenzymatic *cis*-preferential role of 3D, which our data imply has altered RNA binding affinity and plays a role in binding structured RNA elements in the UTR. Our data advance the current understanding of picornavirus replication complex assembly.
